# The Role of Reactive-Oxygen-Species in Microbial Persistence and Inflammation

**DOI:** 10.3390/ijms12010334

**Published:** 2011-01-13

**Authors:** Ralee Spooner, Özlem Yilmaz

**Affiliations:** 1 Department of Periodontology, University of Florida, Gainesville, FL 32610, USA; E-Mail: ralee.e.spooner@gmail.com; 2 Emerging Pathogens Institute, University of Florida, Gainesville, FL 32610, USA

**Keywords:** reactive-oxygen-species, opportunistic pathogens, microbial persistence, extracellular ATP, P2X_7_ receptor, Porphyromonas gingivalis, NLRX1, NADPH oxidase, epithelium

## Abstract

The mechanisms of chronic infections caused by opportunistic pathogens are of keen interest to both researchers and health professionals globally. Typically, chronic infectious disease can be characterized by an elevation in immune response, a process that can often lead to further destruction. Reactive-Oxygen-Species (ROS) have been strongly implicated in the aforementioned detrimental response by host that results in self-damage. Unlike excessive ROS production resulting in robust cellular death typically induced by acute infection or inflammation, lower levels of ROS produced by host cells are increasingly recognized to play a critical physiological role for regulating a variety of homeostatic cellular functions including growth, apoptosis, immune response, and microbial colonization. Sources of cellular ROS stimulation can include “danger-signal-molecules” such as extracellular ATP (eATP) released by stressed, infected, or dying cells. Particularly, eATP-P2X_7_ receptor mediated ROS production has been lately found to be a key modulator for controlling chronic infection and inflammation. There is growing evidence that persistent microbes can alter host cell ROS production and modulate eATP-induced ROS for maintaining long-term carriage. Though these processes have yet to be fully understood, exploring potential positive traits of these “injurious” molecules could illuminate how opportunistic pathogens maintain persistence through physiological regulation of ROS signaling.

## 1. Introduction

One of the early responses of host innate immunity is the Reactive-Oxygen-Species (ROS) production in reaction to microbial invaders. Free oxygen radicals are highly toxic to pathogens and are utilized as a tool to prevent colonization of tissues by microorganisms. ROS are also a key part of the intracellular redox profile influencing a wide variety of signaling networks [1]. Therefore, the relatively high-level of oxidants, as seen in respiratory burst or low-levels in normal tissue, is likely a significant aspect of cell physiology during infection. Oxidative stress occurs when oxygen radical formation and levels exceed those of antioxidants, potentiating cell responses such as apoptosis, tumorgenesis, and immune-response [1–3]. However, the necessity of ROS for immune function could be exploited by “potential pathogens” to reduce host responses in order to enhance survival and colonization in their target host cells. Consistent with this view, characterization of molecular circuitries between the cellular ROS and a host derived small-danger-molecule “extracellular ATP” (eATP), which has lately been recognized as an important physiological signaling mediator that can convert opportunistic microbes into pro-inflammatory pathogens or provide long-term persistence for disease causing pathogens, is of great interest to current research [4,5]. Accordingly, we will first provide a general overview of recent literature on the mechanisms of production of cellular ROS and its role in establishment of microbes in host tissues. Then, we will present the novel subject of purinergic eATP and P2X_7_ coupling mediated cellular ROS and its significance for the microbial persistence and inflammation through examples that have recently become apparent.

## 2. Cellular ROS and Its Implications for Microbial Infections

ROS molecules participate in multi-faceted activities within cells, acting as secondary signaling molecules for inflammation and immune responses, in addition to directly harming microbes that invade tissues [6]. As a hallmark of innate immunity, it is important to understand the necessity of ROS signaling for host responses to challenges presented by microbial invasion. Efficient clearance of colonizing microorganisms is highly dependent on ROS, thus exploration of the mechanisms between oxygen radicals and innate immune responses associated with pathogen clearance has been an intriguing topic for researchers.

### 2. 1. Sources of Intracellular ROS Production as an Early Response

It is well known that ROS production is rapidly elevated during infection, serving to facilitate pathogen clearance as well as contributing to signaling cascades related to inflammation, cell proliferation, and immune responses [7]. Two of the best characterized sources of ROS during host cell- microbe interactions are the membrane associated NADPH oxidase complex and the mitochondrial electron transport chain (Mt-ETC). The Mt-ETC transiently produces radicals as electrons move between the complexes, primarily at Complexes I [8,9] and III [9] and these play key roles in activation of innate immunity.

The connection between innate immune responses, mitochondrial-ROS and microbial infection has been unclear in the past. Recent evidence suggests that a Nucleotide-Binding domain (NBD) and Leucine-Rich Repeat (LRR)-containing family member receptor (NLR), NLRX1, may provide an answer to this elusive question. NLRs, also well known as Nod-like receptors, are intracellular pathogen-associated molecular pattern (PAMPs) receptors, which serve to alert the cell to the presence of intracellular invaders. Though there are many NLRs, only one of these localizes to the mitochondria to facilitate innate immune responses, NLRX1 [10,11]. NLRX1 stimulates production of ROS molecules, which in turn can directly interact with the pathogen as well as act as a secondary signaling molecule [12–14]. Originally identified to utilize the mitochondrion as a platform to mediate antiviral responses [11], NLRX1 now appears to also be important for immune responses to bacterial infections as well. Knowledge of bacterial-mitochondrial interactions are limited, however, a recent review provides a comprehensive overview of bacterial influences upon the mitochondrion, including NLRX1 [15]. NLRX1-induced ROS appears to be critical for bacterial infections, especially in terms of its signaling properties. A study performed using *Shigella*-infected and tumor necrosis factor-α (TNF-α)-primed cells showed that NLRX1 mediated ROS formation [16]. The downstream result of the elevated ROS levels in this particular study was enhanced NF- κB and Jun amino-terminal kinases-dependent (JNK) signaling, demonstrating the role for ROS as a secondary messenger for pro-inflammatory gene transcription and cytokine-based signal responses during infection. Another new study also provided evidence for the key role ROS-mediated signaling can have during infection. This group’s efforts showed that NLRX1 enhanced mitochondrial-derived ROS formation in response to *Chlamydial* infection [17]. Though only a small amount of evidence supports the importance of NLRX1-mediated ROS production during infection, this is most likely due to the recent idea that NLRX1 could be an important mediator of mitochondrial-ROS production during infections. Much like the mitochondria, NADPH oxidase is a primary site for generation of ROS molecules during microbial infection of host cells [18].

NADPH oxidase complexes are common sources of ROS typically associated with rapid respiratory burst mechanisms of professional phagocytic cells, and carry out the electron transferring reaction from NADPH to molecular oxygen (O_2_) in the membranes of phagosomes, endosomes, and the cell membrane [19]. The studies lately have shown the functionally active NADPH oxidase machinery in various cells types including epithelial and endothelial cells [20,21]. NADPH oxidase protein complexes consist of several phox subunits; the cytosolic p40, p47, and p67 in addition to p22 and gp91, which localize to the membrane. The latter two phox subunits, p22 and gp91, make up flavocytochrome b558, the catalytic, superoxide-generating core of NADPH oxidase [22]. A type of Rho-GTPase, called Rac, also associates with the complex [19,22] and mediates NADPH oxidase activity [19,23] and downstream inflammatory signaling processes [24]. Certain regulatory mechanisms are in place to control NADPH oxidase activity, including proteins and wide-sweeping signaling cascades. Control of Rac falls to the Rho-GDP dissociation inhibitor (Rho-GDI), which translocates Rac from the membrane to the cytoplasm, effectively deactivating NADPH oxidase [25,26]. Phosphoinositide-3 kinases (PI3Ks) also manage the NADPH oxidase complex through the modulation of Rac activity and direct binding of the p40/47 subunits [27]. Thus, the powerful induction of ROS and inflammatory signaling cascades by NADPH oxidase are under multiple levels of control, likely to ensure faithful activation of the complex and its downstream signaling intermediates. NADPH oxidase activation is vital for management of microbial invasion, but the precise mechanisms linking activation of the complex with elimination of microorganisms has been not completely characterized. A recent study of LyGDI (a monocyte-specific Rho-GDI [25]) may provide valuable insight into the underlying molecular mechanisms mediating NADPH oxidase activity in response to phagocytosis of microbes. LyGDI was shown to associate with caspase-recruiting domain-containing protein 9 (CARD9) at phagosomes harboring *Listeria monocytogenes* in macrophages [28]. The interaction between CARD9 and LyGDI allowed Rac to associate with the other NADPH oxidase components, thereby providing the activating signal for ROS production by NADPH oxidase. Identifying potential mechanisms for immune response element activation, such as CARD9-lyGDI association, could be crucial to understanding host immunity against microbial invasion.

Thus, the highly regulated NADPH oxidase is a potent contributor to resistance against infection by microorganisms. Due to the key role of NADPH oxidase for participating in immunity, it seems logical that it should be a target for microbial intervention. In actuality, microbes can subvert NADPH oxidase through direct interactions with the phox subunits and Rac. Additionally, mitochondrial-based ROS production can function to eliminate microorganisms. Much like NADPH oxidase, the mitochondrion appears to be a target for microbial interference. We will now provide examples of microbes demonstrated to modulate mitochondrial and NADPH oxidase-induced ROS to mediate immune evasion.

### 2. 2. Modulation of Intracellular ROS by Infection

Immune cells depend on ROS to not only kill phagocytosed microorganisms, but also to mediate inflammatory and immune signaling cascades. There is direct evidence that a wide variety of microbes can limit ROS, thus increasing the potential for persistent infection [29]. This novel strategy to promote microbial survival within the hostile host environment appears to be primarily the result of NADPH oxidase and mitochondrial-derived ROS modulation.

Many bacteria directly interfere with accumulation of ROS molecules via prevention of NADPH oxidase assembly. *Francisella tularensis* live vaccine strain was shown to not only escape neutrophil phagosomes, but also directly prevented NADPH oxidase assembly during intracellular infection [30]. This bacterium specifically prevented recruitment of gp91*^phox^*/p22*^phox^* and p47/p67*^phox^* subunits to the phagosome, thereby attenuating the oxidative burst mechanism utilized by neutrophils. Another bacterium, *Anaplasma phagocytophilum*, prevents p22 and gp91 incorporation into phagosomes containing the microbe, thus attenuating NADPH oxidase activity [31,32]. It is also suggested that *A. phagocytophilum* also directly scavenges ROS, providing an additional defense mechanism against oxygen radical-based host immunity [31].

A study of *Chlamydia trachomatis,* a bacterial pathogen associated with urogenital infections, demonstrated that the rapid induction of ROS within macrophages could be attenuated over a period of time [33]. This was shown to be accomplished by incorporation of the Rac subunit of NADPH oxidase to the *Chlamydia*-containing vacuole, thus inhibiting oxidative burst. The most important observation of this study, however, is that *Chlamydia* can alter the intracellular ROS content back to normal steady-state conditions [33]. By returning ROS levels to normal, as seen in uninfected cells, the *Chlamydia* essentially masks its presence by altering a key immune sensor and inflammation activating mechanism.

The ability to inhibit recruitment of NADPH oxidase subunits appears to be a common theme for many other pathogens, including *Legionella pneumophila* and *Coxiella burnetii. C. burnetii*, which is known to cause chronic Q fever in livestock and humans, prevents recruitment of p47/p67 to the phagosome and thus limits respiratory burst within infected neutrophils [34]. Prevention of p47 recruitment is also a facet of *Legionella* infection of macrophage-like cells [35]. Despite the fact that a strong majority of literature introducing this strategy involves hematopoietic-cell lineages, there is evidence supporting NADPH oxidase assembly interference during infection of other tissue types. For example, *Bacillus anthracis* edema toxin has been implicated in prevention of NADPH oxidase activity [36]. This was shown to be achieved through Protein Kinase A-mediated phosphorylation of the NADPH oxidase component p67, in intestinal epithelium [36].

Extracellular microorganisms also possess the ability to alter intracellular ROS production, contributing to persistence of infection. *Burkholderia cenocepacia* are opportunistic pathogens implicated in chronic inflammatory conditions including Cystic Fibrosis (CF). These bacteria have been demonstrated to inhibit ROS production in infected neutrophils, primarily through exopolysaccharide formation [37]. In addition to interfering with neutrophil chemotaxis, the exopolysaccharides demonstrated the ability to directly scavenge free oxygen radicals, regardless of their intracellular origination. *B. cenocepacia* also has been shown to exist in macrophage vacuoles with delayed NADPH oxidase incorporation, suggesting that it can persist within host cells by prevention of NADPH oxidase components and oxidative burst [38]. In another opportunistic bacterium associated with CF, *Pseudomonas aeruginosa*, secondary metabolite production by the pathogen has been shown to interfere with phagocytic engulfment of apoptotic cells [39]. This secondary metabolite, pyocyanin, mediates this process by reducing intracellular ROS levels and Rho GTPase activities in host cells, likely leading to enhanced inflammation at sites of infection due to failure to phagocytose dying cells [39].

*Neis*s*eria gonorrhoeae* is known to suppress oxidative bursts in neutrophils [40]. This observation was not observed for nonviable *N. gonorrhoeae*, suggesting that live cell metabolic activity directly relates to suppression of oxidative burst. Though this study did not attempt to identify potential targets for inhibition of NADPH oxidase ROS, the observed reduction in oxygen radical production was shown to be dependent upon contact between the host cells and the metabolically active bacteria [40].

*Candida albicans*, an opportunistic fungal pathogen of humans, has the ability to suppress ROS production in host immune cells [41]. The precise mechanism utilized by *C. albicans* remains unclear, but a recent study presented findings indicating that the microorganism is capable of overcoming auto-induction of ROS, resulting in suppressed oxygen radical formation [41]. Another fungal pathogen, *Aspergillus fumigatus*, has been shown to limit ROS production in neutrophils [42,43].

Unlike the previously described organisms, *Entamoeba histolytica*, potentiates NADPH oxidase ROS accumulation in host cells, resulting in apoptosis [44]. This parasite utilizes the secondary signaling properties of NADPH oxidase-derived ROS molecules to influence Extracellular signal-Related Kinase (ERK) 1/2 signaling, eventually stimulating host cell death. Though typically associated with cell survival and proliferation, there is documented evidence supporting the potential for ERK1/2 to induce cell death [45–47]. Infection of host cells by two other pathogens, *Trypanosoma cruzi*, the causative agent of Chagas’ disease, and Japanese encephalitis virus (JEV), also results in enhanced ROS formation. These two pathogens also utilize enhanced ROS to induce host cell death, thus allowing escape and likely contributing to spread of the pathogens through the apoptotic bodies.

JEV infection of promonocytic cells resulted in host cell apoptosis mediated by intracellular accumulation of ROS and activation of pro-apoptotic signaling [48]. Destruction of host cells by virus-induced ROS production could facilitate persistence of viral infection by enhancing the spread of virions. *T. cruzi* can infect humans and lie dormant, only to reemerge and cause chronic inflammatory destruction of organs. Interestingly, a recent study demonstrated that *T. cruzi-*induced ROS accumulation did not involve NADPH oxidase [49]. Further analysis in this report showed that enhanced ROS formation occurred due to pathogen-mediated mitochondrial membrane potential perturbation. Though this was not the first suggestion that *T. cruzi* optimizes infection and persistence through mitochondrial ROS accumulation and oxidative stress [50], this study does demonstrate the current level of interest that researchers have in regards to understanding microbial-mediated influences on ROS production.

Possession of the High Pathogenicity Island (HPI), encoding yersiniabactin, appears to play a role for pathogenesis of *Yersinia* and members of Enterobacteriaceae. A new study suggests that due to the iron-scavenging nature of yersiniabactin, it could provide a novel way to inhibit ROS formation by preventing the Haber-Weiss hydroxyl-radical-forming reaction in infected host cells [51]. Though this may be an indirect method to limit pathogen clearance by host cells, it is evident that bacteria possessing HPI display enhanced pathogenesis [51]. Additional *Yersinia*-related virulence factors have been extensively studied [52–55]. However, more detail studies are required to fully characterize *Yersinia*-directed ROS inhibition, especially for determining the sources of mechanisms providing the ROS regulation.

Modulation of ROS levels appears to be a conserved strategy utilized by various microorganisms. Induction or inhibition of oxygen radicals could facilitate enhanced colonization, via reduced ROS-mediated host responses to infection. Additionally, regulation of cellular ROS by microorganisms can play a critical role in chronic inflammatory diseases and cancer. We will now focus on our main interest of signaling molecules, eATP and its putative receptor P2X_7_ generated ROS and the evidence for microbial modulation of this pathway to enhance microbial survival and persistence in host tissues.

## 3. eATP-P2X_7_ Receptor Signaling in Controlling Infection and Inflammation via Cellular ROS

### 3. 1. eATP as a “Danger Signal”

Adenosine-5′-triphosphate, ATP, is a nucleotide molecule utilized for a variety of processes, including cellular metabolism and signaling. ATP can be released into the extracellular environment by cells under normal conditions as well as a result of cellular stress, infection or cell death. Once outside of the cell, it is identified as extracellular ATP. eATP has become well recognized for its role as a “danger signal” for immune activation [56,57]. Effect of eATP is carried out via stimulation of cellular purinergic receptors and their associated signaling processes. Lately, this host-cell derived small molecule induced responses have become a key topic for researchers studying the pathogenic nature of a wide variety of microorganisms. This may represent a novel target for pathogen-mediated subversion of the host immune response.

The evidence supporting the role of eATP as a potent activator in innate immunity is quite large. Pro-inflammatory cytokine production and secretion is a well characterized event in numerous pathologies, and ATP clearly has a role in initiating these events. For example, eATP has been shown to elicit transcription of IL-6 in macrophages [58]. IL-6 plays an important role in phagocytic cell responses of innate immunity, primarily serving as a mediator of macrophage development from monocytic cells [59]. IL-6 stimulation of monocytes also suppresses dendritic cell (DC) formation, suggesting eATP may perform antagonistic roles in immunity, possibly in immunotolerance [60]. It should also be noted, that eATP has been implicated in maintenance of lung airway inflammation via DC activation [61], thus demonstrating the complex nature of eATP-mediated processes (For a detailed review regarding multifaceted capabilities of ATP on various cell types, readers should see [56] for more information). eATP is also involved in production of the inflammatory cytokine IL-1β, a participant in the regulation of cell differentiation, death, and inflammation. A study performed in 1998 characterized eATP-induced release of IL-1β from LPS-primed monocytes, while also confirming that this process was dependent on the purinergic P2X_7_ receptor [62]. It also appears that in other cell models, such as fetal astrocytes, IL-1β-mediated transcription factor expression is linked with ATP and P2 receptor signaling [63,64]. Elevated Nuclear Factor-kappaB (NF-κB) and activator protein-1 (AP-1) expression were seen when astrocytes were stimulated with eATP, suggesting a role for purinergic signaling in nervous system inflammatory pathologies [64].

Acting as a sensor for pathogens, the inflammasome promotes pro-inflammatory cytokine production, a process dependent upon generation of ROS. The inflammasome is a critical component of the innate immune system and facilitates heightened immune activities against pathogenic invaders, such as *Chlamydia* [65]. The inflammasome consists of Apoptosis-associated speck protein (ASC), caspase-1 and NLR3, NOD-like receptor protein 3 [66]. Cleavage of immature pro-IL-1β by capsase-1, results in mature IL-1β formation [67]. Acting as a secondary signaling molecule, ROS are required for NALP3 inflammasome formation [66] and recent studies demonstrate the necessity of ROS to mediate this process [43]. Furthermore, the processing of IL-1β in *P. gingivalis*-infected primary gingival epithelial cells was shown to require eATP stimulation via P2X_7_ receptor coupling for cytokine release [68]. This could imply that not only can eATP provide a pro-inflammatory stimulus to cells, but it can also directly influence final processing and release of cytokines.

Taken together, eATP signaling exhibits a multi-tiered influence on inflammation via innate immunity. A confounding question, however, is whether innate and adaptive immunity can be linked through eATP signaling and associated ROS production. A recent study of anticancer therapy has identified a role for eATP signaling in directing both innate and adaptive immunity. It is well known that dying and injured cells release ATP, even during anti-tumor treatments [69]. The present study also identified induction of Il-1β secretion from DCs in response to ATP release, resulting in priming of interferon-gamma (IFN-γ)-producing lymphocytes. Thus, eATP has effectively created a potent anticancer response, acting through inflammatory cytokine signaling to stimulate adaptive immunity [70]. Another novel finding linked eATP signaling with antigen presentation to coordinate *Mycobacterial* destruction. eATP and P2X_7_ coupling has been shown to rapidly induce shedding of MHC-II containing exosomes from macrophages as a result of inflammasome activation [71,72]. As we have mentioned before, ROS secondary signaling facilitates inflammasome formation and activity [66], thus ROS can mediate both innate and immune responses. The same group expanded their studies to include *Mycobacterium* infection of phagocytes, and found that the released exosomes contained MHC-II: Mtb Ag 85B (a *Mycobacterial* antigen) [73]. The antigen presenting MHCII:Mtb Ag 85B complexes were also shown to activate T-lymphocytes [73], thus providing an important insight into eATP-P2X_7_-mediated synergy of innate and adaptive immune responses.

It is tempting to speculate that innate and adaptive immunity can be linked to operate concurrently through eATP mediated ROS production. These mechanisms are likely facilitated by the secondary signaling capabilities of ROS, and we have seen clear evidence supporting the role of oxygen radicals in immune activation. However, it is important to recognize the highly complex and dynamic nature of the immune system, and coordination of both the innate and adaptive immunity is not likely to be exclusively limited to eATP purinergic signaling.

### 3. 2. P2X_7_ Receptor Activation by eATP Elicits Signaling Cascades through ROS

Purinergic receptor signaling lately has emerged as an important signaling pathway related to various cellular processes. P2X_7_ has been classically distinguished from the other P2 receptors due to the fact that it is a ligand-gated ion channel and it has low-affinity to eATP concentration. Upon eATP binding of P2X_7_, Ca^2+^ ions flow through the pore formed in the membrane of the cell, creating signal-transducing stimuli. This in turn activates several signaling intermediates, including NADPH oxidase ROS production, which can operate to influence pro-inflammatory and immune response elements, such as cytokine synthesis and transcription factor activation [74–76].

A large body of evidence suggests that the signaling networks of eATP-P2X_7_ coupling are in fact dependent on ROS production. In 1997, it was discovered in microglial cells that NF-κB, a potent pro-inflammatory transcription factor, expression was influenced to a large degree by P2X_7_ [77]. This study also revealed that unlike other NF-κB complex formation resulting from other stimuli, such as LPS, ATP specifically stimulated formation of p65 homodimers. More importantly, the same study demonstrated that formation of NF-κB was abolished when cells were treated with antioxidants, thus providing strong evidence for the necessity of eATP-induced ROS generation for pro-inflammatory gene transcription [77].

Another recently described transcription factor, cyclic-AMP responsive element binding protein (CREB), which plays a protective role for cell survival, appears to be important during elevated ROS conditions (e.g., oxidative stress). Unlike NF-κB, which is associated with pro-inflammatory cytokine production and cell death, CREB protects cells against oxidative stress-induced cell death [78–80]. It also seems that eATP-P2X_7_ coupling can influence the CREB pathway, perhaps offering cells a level of protection against oxidative stress-mediated cell death [79,81]. It remains to be seen how CREB participates in infection models, but it could provide a level of resistance to cell death in response to pathogenic challenge.

Much like the dualistic nature of ROS molecules themselves, the transcription factors that oxygen radicals influence can potentiate antagonistic responses in cells. Though we have already shown evidence for microbial modulation of ROS, primarily via NADPH oxidase interference, eATP-P2X_7_ coupling can also be a target for microorganisms seeking to alter ROS production for their long term carriage. NADPH oxidase complexes are now associated with P2X_7_ signaling cascades, thus interference directly at this receptor could provide another way for microbes to enhance survival and persistence.

### 3. 3. eATP-P2X_7_ Receptor Signaling Mediates Pathogen Killing

There is a noteworthy amount of research regarding eATP-induced pathogen elimination via the P2X_7_ receptor. Toxoplasma *gondii,* for example, has been demonstrated to be eliminated by eATP-P2X_7_ coupled production of ROS molecules, thus facilitating reduction of parasitic load within host cells [82] (Another study yielded similar results, however, this group did not test for ROS involvement in the observed clearance of *T. gondii* [83]). In these two studies, however, *T. gondii*-infected host cells were subjected to rapid eATP-mediated apoptosis, a response serving to likely control extracellular spread of *Toxoplasma* [82,83].

Another microorganism that establishes chronic intracellular infections and is also subject to P2X_7_-mediated elimination is *M. tuberculosis*. Studies of this organism in host cell models clearly provide evidence of eATP-P2X_7_ induced *Mycobacterial* elimination through multiple processes, including apoptosis [84], phospholipase-D [85], autophagy [86] and phagosome-lysosome fusion [87]. Thus, P2X_7_ is likely a major contributor against *Mycobacterial* immunity. In addition, there have been numerous studies suggesting that polymorphisms in the P2X_7_ gene may increase susceptibility to *Mycobacterial* infections, demonstrating the importance of functional P2X_7_ in these pathologies [88–92].

Chlamydial infections of host cells are also found to be limited by eATP-P2X_7_ signaling processes. eATP-P2X_7_ stimulation of Phospholipase-D activity was shown to be required to limit some *Chlamydial* infections of epithelium and macrophages [93–95]. Taken together, these studies show that eATP and P2X_7_ receptor signaling are important immune response elements to a variety of pathogenic infections. However, targeting of eATP-P2X_7_ coupling could be exhibited by pathogens, and in fact there are examples of microbes that exploit this to establish persistence and survival.

### 3. 4. Subversion of eATP-P2X_7_ Pathway by Persistent Microbes

Intracellular parasites can exhibit selective pressures on the P2X_7_ receptor-mediated signaling network, influencing host cell physiology to enhance survival. These effects are likely to be highly variable and dependent on multiple factors, including specific invading organism, host tissue type and physiology, environmental pressures, and even the presence of other colonizing organisms. This exploitation of eATP and the P2X_7_-mediated cell immune response represents a fascinating mechanism of overcoming host attempts to limit infection.

Some evidence suggests direct microbe-mediated modulation of P2X _7._ Studies of *Chlamydia psitacci* show that the Ca^2+^ signal resulting from eATP-P2X_7_ coupling is diminished, resulting in reduced host cell apoptosis [96]. Though we have provided evidence for other organisms exhibiting ROS-modulatory effects to promote persistence, this study shows that P2X_7_ can also be a target to promote microbial survival in host tissues.

*Leishmania amazonensis* also has been demonstrated to interfere with eATP-P2X_7_ mediated signaling and associated ROS generation [97]. Identification of a multi-functional ATP utilizing enzyme ecto-Nucleoside diphosphate kinase (Ndk) in this protozoan has been attributed with eATP scavenging capabilities, reducing eATP-P2X_7_ induced host cell death during infection [98]. Additionally, the supernatant from *M. bovis*, an opportunistic pathogen, containing the Ndk protein decreases the activity of P2X_7_ receptor [99].

*Porphyromonas* gingivalis, a successful host-adapted pathogen capable of intracellular replication and spreading within the host cells, faces the challenge of host-mediated immune responses to clear the microorganism and prevent colonization [100–102]. Similarly, *P. gingivalis* has also been shown to exploit eATP-P2X_7_ signaling associated with host cell death in primary gingival epithelial cells (GECs) [103]. The organism accomplishes this by secreting an Ndk homologue, which hydrolyses eATP, thereby decreasing P2X_7_ ligation, resulting in the inhibition of GEC cell death. Indeed, current studies by our laboratory of *P. gingivalis* indicate that the bacterium is also capable of modulating the cellular ROS levels and protecting primary GECs against eATP-P2X_7_ induced oxidative-stress by inhibiting the global ROS production upon prolonged infection in the host cells. This effect appears to be mediated by both utilizing host NADPH oxidase and mitochondria associated oxidative-stress pathways via time-dependent secretion of *P. gingivalis*-Ndk enzyme during the infection [104]. *P. gingivalis* infection has also been demonstrated to inhibit mitochondrial membrane depolarization, cytochrome *c* release, and activation of caspase 3 and pro-apoptotic Bad through pro-survival PI3K/AKT pathway [105–107]. AKT and Extracellular signal-related kinase (ERK) 1 and 2, members of the mitogen-activated protein kinase (MAPK) network, have been studied in relation to P2X_7_, revealing interesting results. It was shown that ERK1/2 activity during LPS-stimulation of macrophages could be under control of P2X_7_ receptor [108,109]. In ovarian cancer cells, cellular ROS stimulation was found to be important for signal cascades, particularly for AKT and ERK [110]. As mentioned earlier, eATP was demonstrated to be required for release of IL-1β from *P. gingivalis*-infected GECs [68]. The same study also reported that GECs express functional Nalp3 inflammasome formation during the *P. gingivalis* infection, suggesting an additional role for ROS secondary signaling in this infection model.

**Figure 1:**
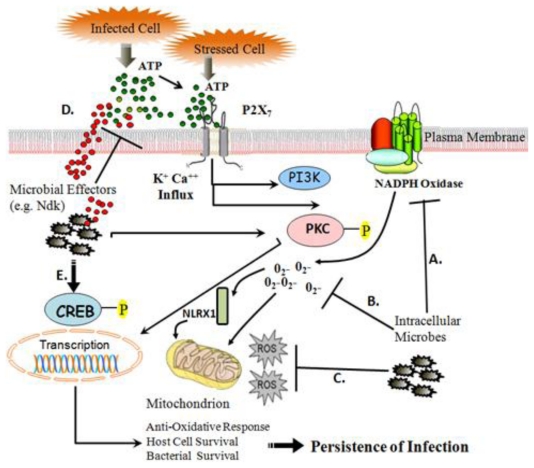
Proposed mechanisms for modulation of intracellular ROS via P2X_7_, NADPH oxidase, and Mitochondrion Interference. (**A**) Invasive microorganisms subvert with NADPH oxidase-based ROS formation via interference of complex assembly; (**B**) Microbial scavenging of ROS produced by NADPH oxidase; (**C**) Interference of mitochondrion-based ROS production during infection; (**D**) Modulation of eATP-P2X_7_ signaling induction by nucleotide scavenging; (**E**) CREB could be involved in promoting host cell survival, and thus may potentiate microbial persistence.

Whether the ROS production and redox signaling are specifically influenced by the above discussed modulatory pathways during *P. gingivalis* infection remains to be seen. However, it is likely that eATP-P2X_7_ mediated ROS signaling play an important role in the persistent nature of this successful opportunist in the epithelial cells. As a model organism, *P. gingivalis*, a major etiological agent in the formation of chronic inflammatory/infectious diseases (namely periodontal diseases) could help explain some fundamental properties of chronic conditions associated with host-adapted opportunistic microbes, perhaps translating to new insights into other chronic inflammatory pathologies (Figure 1).

Intriguingly, a new study illustrated that NLRX1 and NADPH oxidase are both involved in *Chlamydia trachomatis*-induced ROS formation. NLRX1 and the ROS formed in the mitochondria actually promote the bacterial growth [17]. Depletion of NLRX1 and inhibition of NADPH oxidase ROS production attenuated *Chlamydial* load. In previous studies performed by the same group, it was discovered that *Chlamydial* growth was enhanced by ROS-mediated caspase-1 production via inflammasome formation [111]. It now seems that NLRX1 and NADPH oxidase play an important role in generating the ROS required to mediate signaling cascades resulting in caspase-1-dependent growth of *Chlamydia* [17]. It is logical to suggest that eATP can act as a physiological mediator for the cross-talk that takes place between the two pathways and include an additional layer of complexity in the whole host-microbe interaction.

## 4. Conclusions

It is quite notable that a number of distantly related intracellular pathogens exhibit similar abilities to influence inflammation and immune clearance by modulating eATP-P2X_7_ derived cellular ROS. This novel occurrence appears as a common theme that enhances survival of both the host and the invading microorganism, and it can be achieved through reduction of intracellular ROS through exploitation of host metabolic pathways by host-adapted microbes. More intriguingly, successful colonizers may also “unwittingly” have come to use physiological ROS signaling properties for their own designs including microbial utility of eATP induced ROS as growth signaling molecules. Overall, a large amount of recent evidence suggests that a wide array of pathogens have developed various complex, yet converging molecular strategies to limit or exacerbate ROS formation in order to subvert immune defenses and support self-survival and long term carriage in their target host cells.
